# Gut Microbiota of Chinese Obese Children and Adolescents With and Without Insulin Resistance

**DOI:** 10.3389/fendo.2021.636272

**Published:** 2021-03-19

**Authors:** Xin Yuan, Ruimin Chen, Ying Zhang, Xiangquan Lin, Xiaohong Yang, Kenneth L. McCormick

**Affiliations:** ^1^ Department of Endocrinology, Genetics and Metabolism, Fuzhou Children’s Hospital of Fujian Medical University, Fuzhou, China; ^2^ Division of Pediatric Endocrinology and Diabetes, University of Alabama at Birmingham, Birmingham, AL, United States

**Keywords:** insulin resistance, gut microbiota, obesity, children, ANGPTL4

## Abstract

**Objective:**

The intestinal flora of gut microbiota in obese Chinese children and adolescents with and without insulin resistance (IR) was analyzed, as well as associations between the gut microbiota and two serum cytokines related to glucose metabolism, adropin and angiopoietin-like 4 (ANGPTL4).

**Methods:**

Clinical data, fecal bacterial composition, glucose-related hormones, and serum adipokines (adropin and ANGPTL4) were analyzed in 65 Chinese children with exogenous obesity. The composition of the gut microbiota was determined by 16S rRNA-based metagenomics and IR was calculated using the homeostasis model assessment (HOMA).

**Results:**

The 65 obese subjects were divided into two groups: insulin sensitive (IS) (n=40, 57.5% males) or IR (n=25, 60% males). Principal coordinates analysis revealed that the gut microbiota samples from the IS group clustered together and separated partly from the IR group (p=0.008). By Mann-Whitney *U*-test, at a phylum level, a reduction of *Firmicutes* and an increase of *Bacteroidetes* in the IR subjects was observed. LEfSe analysis revealed that IS subject, when compared to their IR counterparts, harbored members of the order *Coriobacteriales*, *Turicibacterales*, *Pasteurellales* and family *Turicibacteraceae*, that were significantly more abundant. In contrast, the IR subjects had members of family *Peptococcaceae* that were significantly more prevalent than the IS subjects (all *p*<0.05). Spearman’s correlation analysis revealed that serum ANGPTL4 was positively associated with genus *Bacteroides, Butyricimonas*, and *Alistipes*, and adropin was positively associated with genus *Anaerostipes* and *Alistipes*, and negatively associated with genus *Blautia* (all p<0.05).

**Conclusion:**

In obese children, the gut microbiome in IR subjects was significantly discordant from the IS subjects, and the abundance of some metabolism-related bacteria correlated with the serum concentrations of adropin and ANGPTL4. These observations infer that the gut microbiota may be involved in the regulation of glucose metabolism in obesity.

## Introduction

The worrisome prevalence of obesity among children, adolescents, and adults is arguably one of the most serious public health concerns worldwide given the proclivity for cardiovascular diseases and type 2 diabetes (T2DM) (1). Insulin resistance (IR) is a cardinal metabolic feature of T2DM, and it is also the pathophysiological underpinning of various metabolic diseases in obesity, such as polycystic ovarian disease and hyperlipidemia. According to some studies, the major cause of IR in obesity is aberrant cellular lipid partitioning pattern characterized by increased deposition of lipids within insulin-responsive tissues, such as the liver and skeletal muscle. This divergent lipid accumulation is also associated with infiltration of intra-abdominal cells of the immune system, which induces a systemic, low-grade inflammation (2).

In adults, gut microbiota dysbiosis has been proposed as an etiologic factor underlying metabolic disease associated with IR, such as obesity and T2DM (3–6). To illustrate, following fecal microbiota transplantation from lean human donors to obese recipients with metabolic syndrome, a significant improvement in insulin sensitivity resulted (7, 8). Furthermore, metagenomic analysis revealed that the gut microflora of T2DM patients versus healthy individuals are disparate- for example, a diminution in butyrate-producing bacteria may impair glucose metabolism in T2DM (9, 10). Gut microbiome can influence host insulin sensitivity by interacting with dietary components and habits (11), and by fermentation production, or lack thereof, of relevant carbohydrate intermediary metabolites (12). Moreover, gut microbiota dysbiosis may beget a pathophysiological mechanism for systemic inflammation in IR (13, 14). Notwithstanding the recent scrutiny of the intestinal flora in numerous disorders, little is known about the characteristics of the gut microbiota in children, let alone those who are obese (15).

Adipokines are critical signaling molecules involved in controlling whole-body energy homeostasis. Angiopoietin-like protein 4 (ANGPTL4), a multifunctional adipokine signal protein expressed in many tissues, may be involved in the regulation of multiple physiological processes, including energy metabolism, glucose homeostasis, fat storage, and lipid metabolism (16). Another adipokine assessed was adropin, which in humans may be associated with energy homeostasis, glucose and lipid metabolism, and insulin sensitivity (17). Our previous research found that serum adropin level was associated with serum insulin level and homeostasis model assessment of insulin resistance (HOMA-IR). Recent studies suggest that the gut microbiota can participate in the regulation of energy metabolism by regulating the levels of adipokines (18). This study determined the characteristics of gut microbiota in obese Chinese children and adolescents with/without IR, and analyzed the association between gut microbiota and circulating cytokines which impact glucose metabolism.

## Patients and Methods

### Study Population

The cross-sectional study consisted of participants managed at Fuzhou Children’s Hospital of Fujian Medical University from September 2017 to March 2018. The study was limited to children who met the following criteria: (a) 5 to 15 year-old, (b) diagnosed as obese according to Chinese criteria (19), and (c) residence of Fujian province.

The exclusion criteria were as follows: any endocrine disease associated with obesity (Cushing syndrome, hypothyroidism, corticosteroid usage, etc.), antibiotic therapy history, gastro-intestinal-related medication, probiotics, or any chronic gastrointestinal or recent diarrheal disease (World Health Organization definition) within 1 month prior to the study.

### Dietary Assessment

In order to appraise dietary habits during the preceding month, a semi-quantitative food frequency questionnaire was developed according to the dietary habits of South China, and this was completed by all participants (20).

### Clinical Assessment

Height and weight were measured by trained nurses. BMI-Z scores were calculated based on Li Hui et al’s reference values (19). Waist and hip circumference were measured as previously described (20). A waist-to-hip ratio (WHR) was calculated by waist circumference (cm) divide by hip circumference (cm), and waist-to-height ratio (WHtR) was calculated by waist circumference (cm) divide by height (cm).

### Laboratory Evaluation

All participants maintained their usual dietary intake at least 3 days, and then fasted for 12 h before a blood sample was obtained. After centrifugation of the blood, serum was stored at −80°C and analyzed within 2 weeks Fasting plasma glucose (FPG) was measured by standard methods (Beckman Coulter AU5800, USA) and fasting insulin (INS) by a chemiluminescent immunoassay (IMMULITE 2000, Siemens Healthcare Diagnostics Products Limited, Germany) using specific reagents. The serum adropin level was assayed using a commercial ELISA kit (Phoenix Pharmaceuticals Inc. USA), with inter-assay and intra-assay coefficients of variation (CV) less than 10% and 15%, respectively. Serum ANGPT4 levels was measured using a commercial ELISA kit (Item number: ab99974, Abcam, UK), with detection limit <20 pg/ml. Fecal samples were collected and processed as previously described (20).

### Definition of IR

HOMA-IR was calculated using the following formula: INS (mIU/L) ×FPG (mmol/L)/22.5. A HOMA-IR value >4 is considered IR, and values ≤4 deemed insulin sensitive (IS) (21, 22).

### Genomic DNA Extraction and Library Construction

The microbial community DNA was extracted and quantified as previously described (20). Variable regions V3-V4 of bacterial 16s rRNA libraries was prepared and library construction was performed as previously described (20). The operational taxonomic units (OTUs), defined by a 99% similarity threshold, were chosen and the representative sequences were submitted to GreenGenes (v 13.8) for the sequence alignment.

### Statistical Analysis

Statistical analyses of clinical data were performed using the Statistical Package for the Social Sciences software version 23.0 (SPSS Inc. Chicago, IL, USA). The normality of the data was tested using the Kolmogorov-Smirnov test. Depending on the data distribution, data are expressed as mean ± SD or median (25th–75th percentiles), and comparisons of the results were assessed using independent samples t test, Mann-Whitney U test and Kruskal-Wallis test. Comparison of rates between two groups was by chi-square test. Spearman’s correlation was used to analyze the relationship between relative abundance of gut microbiota and serum cytokine levels, and partial correlation analysis corrected for potential confounders. A value of *P* < 0.05 was considered statistically significant.

Statistical analyses of 16s rDNA sequencing data were performed on alpha- (reflecting intra-individual bacterial diversity) and beta- (reflecting inter-individual dissimilarity) diversity measurements by software QIIME2(v2019.7) (23). Alpha-diversity indices contained the Shannon diversity index (calculates richness and diversity using a natural logarithm), observed OTUs, Faith’s Phylogenetic Diversity (measures of biodiversity that incorporates phylogenetic difference between species) and Pielou’s evenness (measure of relative evenness of species richness). Beta-diversity indices contained Jaccard distance, Bray-Curtis distance, unweighted Unifrac and weighted Unifrac using principal coordinates analysis (PCoA). Kruskal-Wallis Test was used for two group comparison. The *Firmicutes/Bacteroidetes* (*F/B*) ratio was also calculated. Linear discriminant analyses Effect Size (LEfSe) were determined by software LEFSE (24). To predict metagenome functional content from 16S rRNA gene surveys, Picrust2 (25) was applied to obtain the KEGG (Kyoto Encyclopedia of Genes and Genomes) pathways and STAMP (26) was availed to analyze the differential pathway.

### Statistical Power Analysis

We performed post-hoc power calculation for the comparison of the microbiome between IS and IR subjects, focusing on the power of differential abundance analysis. We used the web-based microbiome power calculator to conduct power analysis (http://fedematt.shinyapps.io/shinyMB/), which was based on Monte Carlo simulations and Wilcoxon–Mann–Whitney test (27, 28). We used a false discovery rate of 5% to correct for multiple testing. In this calculator, the WMW test is applied to each OTU individually and the resulting P-values are multiplicity adjusted with the Benjamini and Hochberg (1995) method (29), to control for False Discovery Rate. Assuming that we are testing 62 genera with the abundance of five genera decreasing by 67% in the IR group compared to the IS group, we had an average power of 85% to detect these five differential genera at current sample size. Therefore, the study was reasonably powered to detect a moderate taxa difference when comparing the IS and IR subjects.

## Results

### Study Participants

The age of the 65 participates ranged from 6 to 14.2 years with a mean of 10.0 ± 1.8 years. There were 40 children with IS and 25 children with IR. The age of the IR group was significantly higher than the IS group (p = 0.004). There were no differences in gender, dietary habits, BMI-Z, WHR, WHtR, FPG, fasting INS, serum adropin and ANGPLT4 between the two cohorts (all p>0.05, **Table 1**).

**Table 1:** Anthropometric profiles and laboratory measurements in IS and IR subjects.

	IS (n = 40)	IR (n = 25)	t (Z)value	*p* value
Age (yr)	9.80 (7.83, 11.12)	10.50 (9.85, 11.99)	−2.387	0.017
Male (%)	57.5	60	(0.040)	1.000
BMI (kg/m^2^)	24.71 ± 3.16	26.73 ± 3.89	−2.578	0.012
BMI-Z	2.61 (2.25, 3.15)	2.62 (2.44, 3.19)	−0.701	0.483
WHR	0.88 ± 0.05	0.89 ± 0.05	−0.751	0.455
WHtR	0.53 ± 0.04	0.54 ± 0.04	−0.656	0.514
FPG (mmol/l)	4.89 (4.67, 5.13)	4.90 (4.55, 5.33)	−0.007	0.995
Fasting INS (μIU/ml)	11.48 (9.02, 14.00)	25.70 (23.80, 31.95)	−6.742	<0.001
HOMA-IR	2.59 (1.92, 3.13)	5.48 (5.28, 7.10)	−6.742	<0.001
adropin (ng/ml)	2.43 ± 0.45	2.58 ± 0.66	−0.962	0.342
ANGPTL4 (µg/ml)	0.007 (0.001,1.861)	3.32 (1.22, 7.49)	−1.628	0.104

BMI, body mass index; BMI-Z, BMI standard deviation Z score; WHR, waist-to-hip ratios; FPG, fasting plasma glucose; INS, insulin; HOMA-IR, homeostasis model assessment of insulin resistance; ANGPTL4, angiopoietin-like protein 4. Data are expressed either as mean ± SD or median (25th–75th centiles).

### Microbiota Profiles in IS and IR Subjects

A total of 918,578 sequencing reads were obtained from 65 fecal samples, with an average value of 14,217 counts per sample. We identified an overall of 146 OTUs, among which 136 OTU with ≥2 counts, and they were grouped in 10 phylum and 38 families.

#### Abundance Profiling in IS and IR Subjects

**Figure 1:**
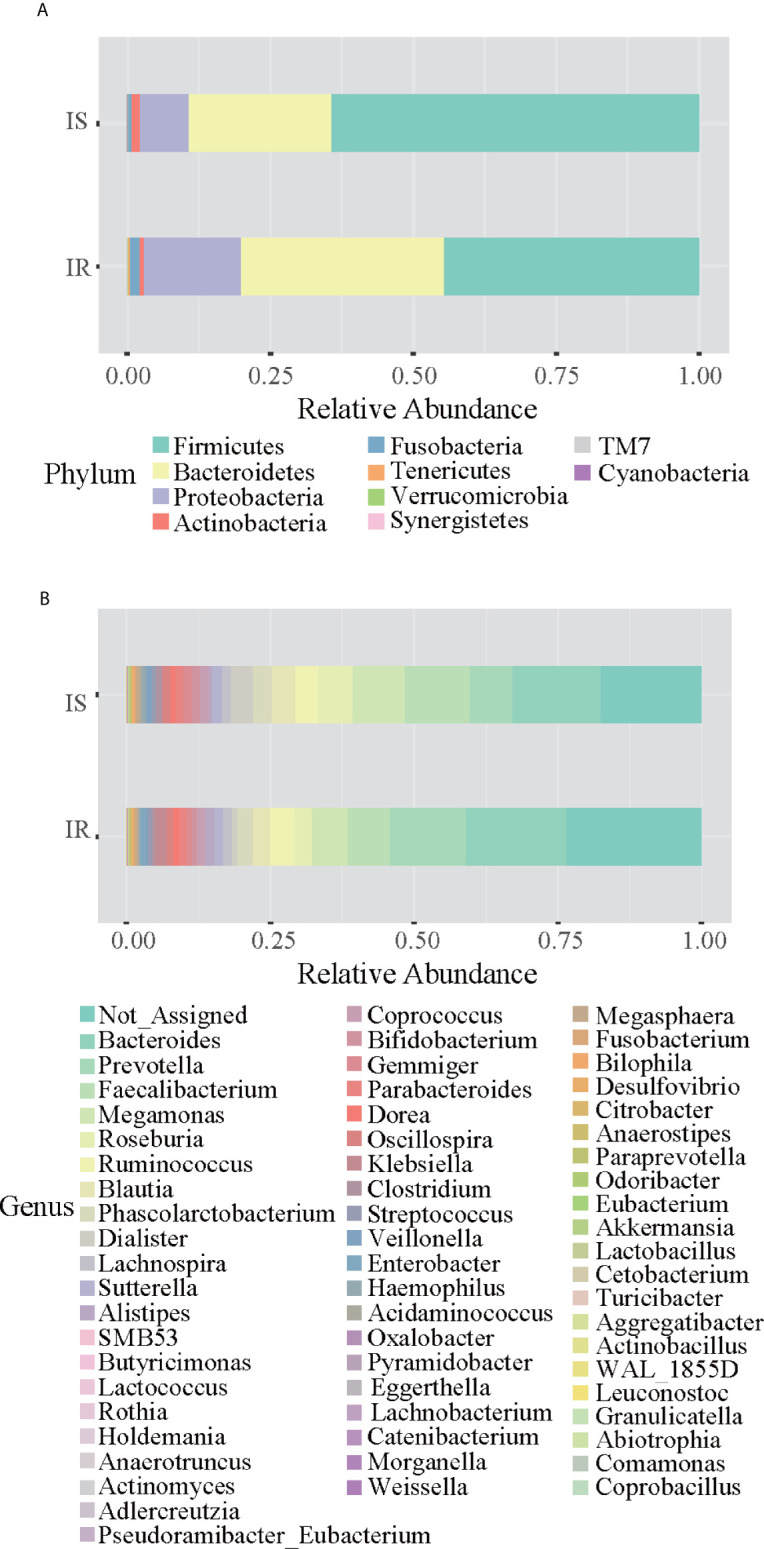
Bar chart representing Mann-Whitney U-test results on operational taxonomic units (OTUs) grouped in phyla **(A)** and in genus **(B)** of the IR and IS groups. Each column in the plot represents a group, and each color in the column represents the percentage of relative abundance for each OTU.

Grouping OTUs at phylum level, and applying the Mann-Whitney U test on the relative abundances of phyla for the two groups, a reduction of *Firmicutes* and an increase of *Bacteroidetes* in the IR subjects was observed compared to the IS subjects (both p <0.05, **Table 2** and **Figure 1A**). Thus, the ratio of *Firmicutes* to *Bacteroidetes* (*F/B* ratio) was significantly higher in the IS group compared with the IR group (p=0.007).

**Table 2:** The mean relative abundance of gut microbiota in IS and IR subjects at phylum level.

	IR	IS	Z	P value
*p_Actinobacteria*	0.010	0.017	−1.423	0.155
*g_Adlercreutzia*	0.000	0.000	−2.394	**0.017**
***p_Bacteroidetes***	**0.357**	**0.252**	−**1.996**	**0.046**
*p_Cyanobacteria*	0.000	0.000	−1.391	0.164
***p_Firmicutes***	**0.446**	**0.643**	−**4.194**	**<0.001**
*g_Anaerostipes*	0.001	0.002	−2.007	**0.045**
*g_Dialister*	0.011	0.039	−2.085	**0.037**
*g_Turicibacter*	0.000	0.001	−2.402	**0.016**
*p_Fusobacteria*	0.017	0.004	−0.032	0.974
*p_Proteobacteria*	0.168	0.083	−0.081	0.936
*g_Haemophilus*	0.003	0.006	−2.262	**0.024**
*p_Synergistetes*	0.000	0.000	−0.78	0.436
*p_Tenericutes*	0.002	0.000	−1.83	0.067
*p_TM7*	0.000	0.000	−0.846	0.397
*p_Verrucomicrobia*	0.000	0.001	−1.312	0.189

Assuming that the total amount of all gut microbiota in each individual is 1, the mean relative abundances of gut microbiota in each group is reported in columns. p_: at phylum level; g_: at genera level. Only the genera whose relative abundance was significantly different in IS and IR subjects are shown in the table. Bold values: p < 0.05.

On OTUs at the genera level, by Mann-Whitney U-test, including all the genera (merging small taxa with counts<10), we found that genera *Adlercreutzia, Anaerostipes, Dialister, Haemophilus*, and *Turicibacter* were more prevalent in IS obese than those who were IR (all *p* < 0.05; **Figure 1B**, **Table 2**).

#### Alpha- and Beta-Diversity in IS and IR Subjects

To assess the overall differences of microbial community structures in IS and IR subjects, we measured ecological parameters based on alpha-diversity. There was no difference of alpha-diversity between IR and IS groups (all p>0.05, **Table S1**).

**Figure 2:**
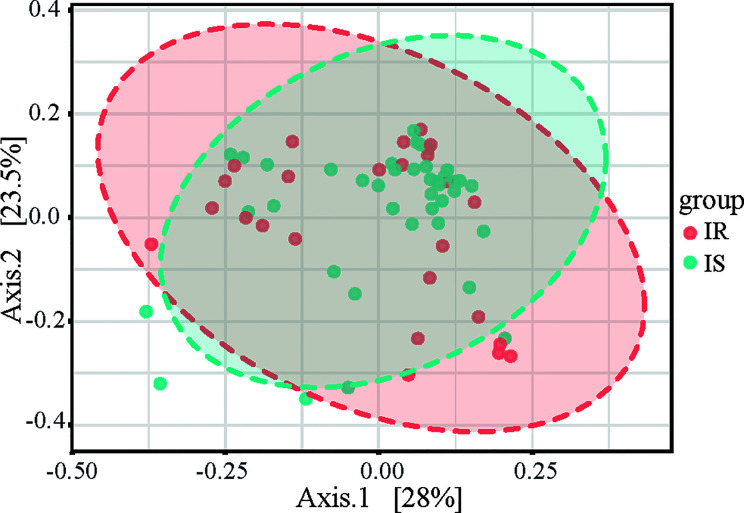
Principal coordinates analysis (PCoA) plot of IR and IS subjects. The plots show the first two principal coordinates (axes) for PCoA using the Bray-Curtis Distance method.

To determine the differences between microbial community structures in IS and IR subjects, we calculated β-diversity. By Distance method Bray-Curtis and PCoA analysis, the gut microbiota samples from the IS group were clustered together and separated partly from the IR group, and insulin sensitivity status explained 28% of the variance in microbiota composition (P = 0.008, **Figure 2**).

#### Bacterial Taxa Differences in IS and IR Subjects

**Figure 3:**
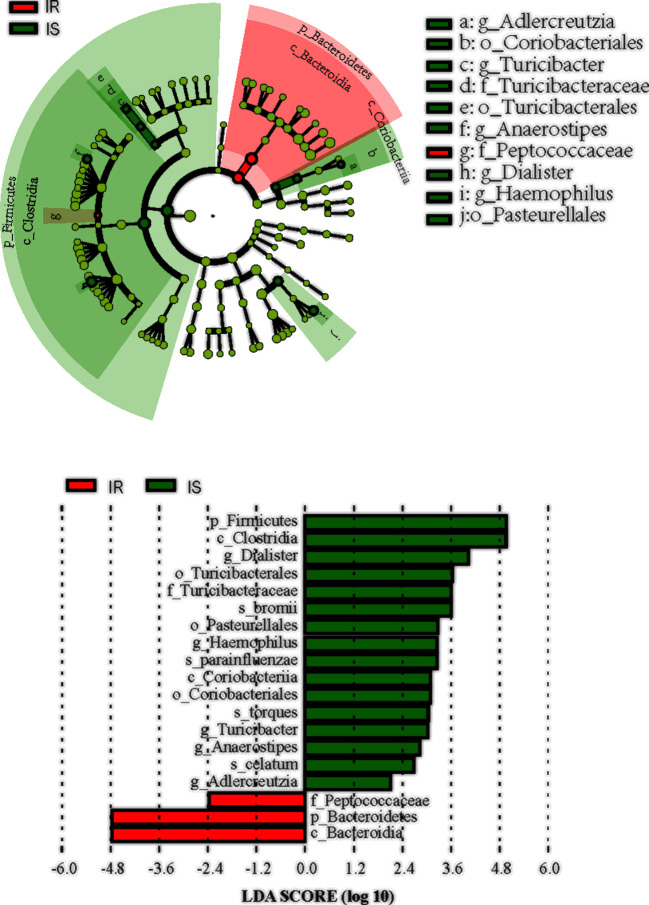
Differential biomarkers associated with genders in IR and IS groups. A linear discriminant effect size (LeFse) analysis were performed (α value = 0.05, logarithmic LDA score threshold = 2.0).

We next used LEfSe analysis to identify bacteria where the relative abundance was significantly increased or decreased in each phenotypic obese category. IS obese children harbored members of the order *Coriobacteriales*, *Turicibacterales*, *Pasteurellales*, family *Turicibacteraceae*, that were significantly more prevalent than IR subjects. In contract, the IR subjects had members of the family *Peptococcaceae* that were significantly more prevalent than the IS subjects (all *p*<0.05, **Figure 3**).

### Detecting Microbial Biomarkers in Both Groups

Discriminant analysis (DA) based on univariate ANOVAs, Fisher’s coefficient and leave-one-out classification were performed to define a model based on the capability of OTUs to discriminate the two groups of study participants (IR and IS subjects).

By DA, at the phyla level, 73.8% of the original grouped subjects were correctly classified by the relative abundance of *Firmicutes* (**Table S2**), and at the genus level, 86.2% of the original grouped subjects were correctly classified, with the genus *Abiotrophia, Adlercreutzia, Aggregatibacter, Anaerostipes, Dialister, Megamonas, Odoribacter, Roseburia*, and *Veillonella* enter the function (from a Wilks’ Lambda test, all P < 0.05). Furthermore, applying a cross-validation test, we found that 81.5% of cases were correctly classified, confirming a high capability of the entire OTUs set to discriminate the two groups (**Table S3**).

### Correlations Between Novel Cytokine Related to Glucose Metabolism and Bacterial Abundance

To evaluate correlations between bacteria and serum cytokine related to glucose metabolism (adropin and ANGPTL4), Spearman’s rho cut-off values were adopted taking into account r > 0.4, r < −0.4 (p < 0.05, **Table S4**). Spearman’s correlation analysis revealed that adropin positively correlated with genus *Anaerostipes* and *Alistipes*, and negatively associated with genus *Blautia*, and ANGPTL4 negatively correlated with the *F/B* ratio and genus *Bacteroides, Butyricimonas* and *Alistipes* (all p<0.05, **Table 3**).

**Table 3:** Spearman’s correlation table on OTUs and cytokine.

	ANGPTL4		adropin	
	R	P value	R	P value
*Bacteroides*	.547**	<0.001		
*Blautia*	−0.415**	0.001	−.412**	0.001
*Actinomyces*	−0.360**	0.004		
*Turicibacter*	−0.355**	0.004		
*Parabacteroides*	0.340**	0.006		
*Eggerthella*	−0.334**	0.007	−.276*	0.028
*SMB53*	−0.336**	0.007		
*Bifidobacterium*	−0.325**	0.009	−.300*	0.017
*Butyricimonas*	0.328**	0.009		
*Streptococcus*	−0.315*	0.012		
*Dorea*	−0.312*	0.013		
*Faecalibacterium*	0.305*	0.015	.259*	0.041
*Weissella*	−0.289*	0.022		
*Leuconostoc*	−0.273*	0.03		
*Adlercreutzia*	−0.273*	0.031		
*Bilophila*	0.271*	0.032		
*Abiotrophia*	−0.251*	0.048		
*WAL_1855D*	−0.249*	0.049		
*Anaerostipes*			.313*	0.012
*Eubacterium*			−.261*	0.039
*Alistipes*			.251*	0.048
*F/B* ratio	−0.432**	<0.001		

OTU, operational taxonomic unit. *P < 0.05; **p < 0.01.

After partial correlation analysis to adjust the impact of BMI, WHR, and ANGPTL4 was still positively associated with genus *Bacteroides, Butyricimonas* and *Alistipes* (r=0.268,0.600 and 0.361, p=0.040,<0.001 and 0.005, respectively), and adropin remained positively associated with genus *Anaerostipes* and *Alistipes* (r=0.384 and 0.290, p=0.007 and 0.026, respectively), and negatively associated with genus *Blautia* (r = −0.273 and p=0.037).

### Metabolic Pathway Predictions

**Figure 4:**
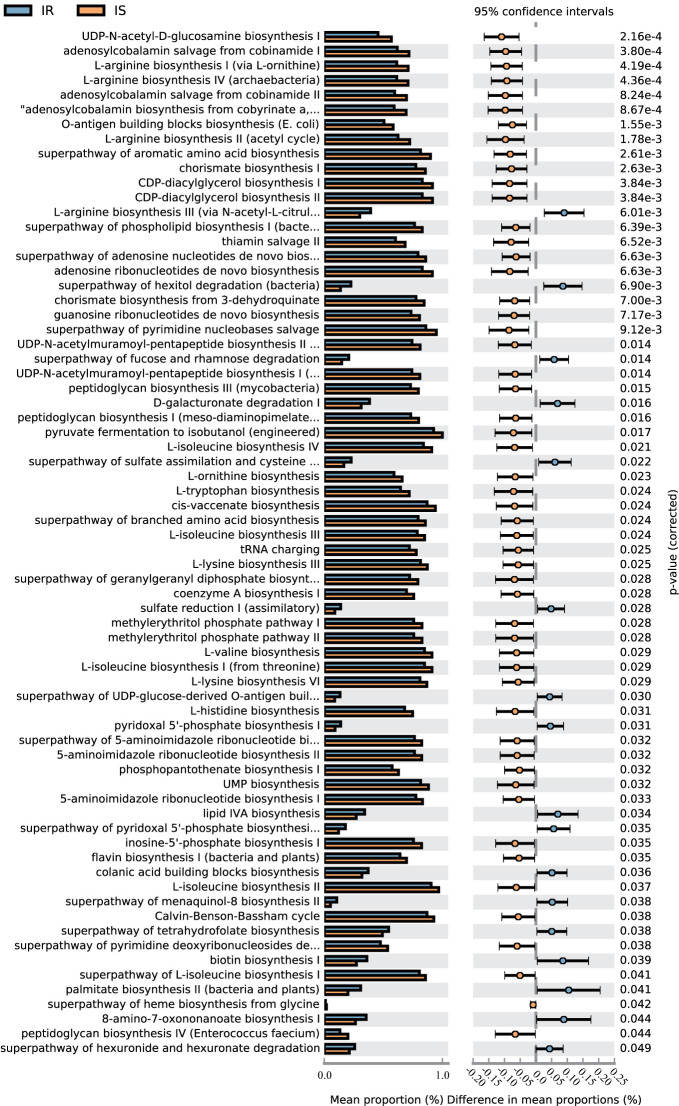
KEGGs biomarkers of IR and IS children.

A total of 70 KEGG pathways were generated using the composition of the gut microbiota based on PICRUSt2 in IR versus IS subjects (**Figure 4**, **Table S4**).Importantly, the glucose metabolism pathways including Calvin-Benson-Bassham cycle, coenzyme A biosynthesis I, peptidoglycan biosynthesis I (meso-diaminopimelate containing), peptidoglycan biosynthesis III (mycobacteria), peptidoglycan biosynthesis IV (Enterococcus faecium) and UDP-N-acetyl-d-glucosamine biosynthesis I were increased in IS subjects and, conversely, the superpathway of fucose and rhamnose degradation, superpathway of hexitol degradation (bacteria), superpathway of hexuronide and hexuronate degradation, and superpathway of UDP-glucose-derived O-antigen building blocks biosynthesis were decreased in the IS subjects (all p<0.05). Furthermore, some nucleotide metabolism pathways (e.g., 5-aminoimidazole ribonucleotide biosynthesis, pyrimidine nucleobases salvage), and amino acid metabolism (e.g., l-arginine biosynthesis, histidine biosynthesis, ornithine biosynthesis) were increased in IS subjects. Finally, in the IR children, some pathways associated with carbohydrate metabolism (d-galacturonate degradation I, hexuronide and hexuronate degradation), amino acid metabolism (e.g., l-arginine biosynthesis III) and lipid metabolism (lipid IVA biosynthesis) were increased (P<0.05).

## Discussion

The gold standard technique to evaluate IR is the hyperinsulinemic euglycemic clamp, however, it is costly and impractical to perform in the clinical setting. Therefore, the HOMA-IR calculation was applied to evaluate IR in the present study given that it correlates favorably with the hyperinsulinemic-euglycemic clamp and it has been validated in children (30). Innumerable studies have confirmed that obese individuals are prone to developing IR and T2DM (31). Herein, we delineated obese subjects according to their insulin sensitivity status, yet there were no significant differences in BMI-Z scores between the two cohorts.

Recent studies of the human microbiome have corroborated the interplay of gut microbiome and disturbed metabolism, and perhaps playing a causative role in the development of obesity, IR and T2DM (32–34). Altering the human gut microbiota may prove useful in developing safe and inexpensive promising therapeutic interventions (35). A plethora of microbiota animal studies notwithstanding, information on intestinal bacterial composition and function in children is limited (36, 37).

Our results confirmed the gut microbiota dysbiosis in obese children with IR, and also characterized bacteria specific to IR. Although no significant difference of alpha-diversity was observed between the two groups, a difference in beta-diversity bacterial communities prevails in obese children with IR.

*Bacteroidetes* and *Firmicutes* occupy the dominant position in human intestine and serve essential roles in nutrient absorption, mucosal barrier fortification, xenobiotic metabolism, angiogenesis, and postnatal intestinal maturation (38). Relevantly, we report a bacterial profile associated with IR in which there was a reduction of *Firmicutes* and an increase of *Bacteroidetes* at phylum level. Furthermore, phylum *Firmicutes* appears to be a microbial marker for IR subjects. Analogous results in relation to these phyla have been found in both humans (9, 39, 40) and mice (41) with T2DM. Compared with the colonization of “lean microbiota” in germ-free mice, the relative abundance of *Firmicutes* was enhanced by the colonization of “obese microbiota” that lead to an amassment in body fat (42). This infers an increased capacity to extract energy from nutrients if the gut is colonized by obesity-associated microbiota. The greater metabolic diversity found in *Firmicutes* with respect to *Bacteroidetes* (348 metabolic pathways versus 76, respectively) also supports the likelihood that the above-mentioned inference is indeed due to the phylum *Firmicutes* (43).

The intestinal *F/B* ratio is a commonly evaluated in metabolic disorders, such as obesity (44). Interestingly, a recent study in humans found a marked dysbiosis, characterized by an increased F/B ratio, in obese metabolic syndrome compared with comparably obese without metabolic syndrome and non-obese (45), suggesting that the F/B ratio may be related to the presence or absence of metabolic traits rather than to obesity itself. However, another study reported that the *F/B* ratio was significantly lower in T2DM patients than in non-diabetic patients, and negatively associated with plasma glucose concentration (40), an observation which is corroborated in our study. Overall, more studies are warranted which consider the manifold cofounding factors, including geography, age, sex, hormonal status, host genetics and diverse diets (46)- all of which may potentially alter the composition of the gut microbiota- in order to assess the relevance of the microbial *F/B* ratio in childhood obesity. Lastly, given the potential impact of diet on the gut microbiome, this study was limited insofar as the diverse dietary habits across China could not be mirrored.

Ussar S et al. opined that members of the *Firmicutes*, such as the genus *Roseburia*, correlated with components of the metabolic syndrome (6). In this study, we found that genus *Roseburia* could correctly classify 86.2% of the original grouped subjects. In humans, gut members of the genus *Roseburia* negatively correlated with plasma glucose (40) and are less gut abundant in individuals with T2DM (47). The abundance of *Roseburia* increase in the feces of obese individuals with metabolic syndrome who receive fecal transplants from lean metabolically healthy donors, and this strategy improved peripheral insulin sensitivity (12). Butyrate produced by *Roseburia* fermentation provides a carbon source for colonic epithelial cells and enhances mitochondrial function in peripheral tissues, yet the exact biochemical mechanisms underlying the salutary metabolic effects of butyrate are uncertain (48).

Chronic, low-grade inflammation is a prevailing characteristic of obesity and T2DM, and this systemic inflammatory response is also thought to drive IR (12). ANGPTL4, an inflammation-related adipokine, may be involved in the regulation of multiple physiological processes including energy metabolism and glucose homeostasis (16). Although circulating ANGPTL4 levels were elevated in diabetes and correlated with glucose levels and HOMA-IR, but not BMI (49), which was evidence for a role for chronic, occult, low-grade inflammation (50), there was no difference in the serum ANGPTL4 concentration in obese children with IR compared with controls in this study. Furthermore, we found the level of ANGPTL4 was positively associated with genus *Bacteroides, Butyricimonas*, and *Alistipes*. It has been reported that the relative abundance of genera *Bacteroides* and *Butyricimonas* were increased with an improvement of insulin signaling and decrease in blood glucose (51, 52) in animal and human studies, our research provides evidence for the same in children. Furthermore, our results showed that adropin was positively associated with genus *Anaer*ostipes and *Alistipes*, and negatively associated with genus *Blautia*. As previously reported, high levels of *Alistipes* and low levels of *Blautia* were also found in patients with diabetes (53, 54). These results indicate that the composition of the gut microbiota is closely related to the levels of blood glucose as well as gluco-metabolic related cytokines, which cause low grade inflammation. Insulin resistance may be a consequence of dysregulation of bacterial production of butyrate, short-chain fatty acids and other metabolites (53).

Compelling evidence has implicated a role for peptidoglycan for the IR, metabolic inflammation and liver disorders (55). In our study, several pathways associated with peptidoglycan biosynthesis were attenuated in the IR subjects. And, in obese subjects with hyperinsulinemia and dyslipidemia, the translocation of peptidoglycan was linked to plasma lysozyme, which was negatively correlated with HOMA-IR, HbA1c, and cholesterol (56). Considering that HOMA-IR is a more rigorous proxy for liver IR versus peripheral IR (57), studies using a hyperinsulinemic euglycemic clamp could determine the role of peptidoglycan in IR obese children.

This cross-sectional study revealed diverse gut microbiota in obese children of different insulin sensitivity statuses. As reported, the intake of artificial sweeteners did not investigate in this study, which may cause IR and effect on the microbiota (58). Furthermore, it is speculated that the gut microbiota may be more sensitive and change earlier than cytokines such as ANGPTL4 and adropin. The correlation between gut microbiota and ANGPTL4/adropin suggests that ANGPTL4 and adropin may still be in a compensatory state and no metabolic disturbance has yet to manifest. A longitudinal study wherein the participants are followed over an extended period (transition from IS to IR to T2DM) would confirm a dynamic change in gut microbiome and cytokines with different glucose metabolism statuses, and could establish causality.

## Conclusion

This study provides evidence of a tripartite interaction between gut microbiota, host immune system and glucometabolic pathways and this could partake in the pathophysiology of obesity and IR. The gut bacteria microsystem in obese IR subjects was significantly different compared with obese IS children: the *F/B* ratio was significantly higher in the IS group compared with the IR group. The abundance of metabolism-related bacteria such as *Anaer*ostipes, *Alistipes*, and *Blautia* was related to the level of ANGPTL4 or adopin, which buttresses the notion that the gut microbiota can moderate glucose metabolism.

## Data Availability Statement

The datasets presented in this study can be found in online repositories. The names of the repository/repositories and accession number(s) can be found below: https://bigd.big.ac.cn/gsa, CRA003010.

## Ethics Statement

The studies involving human participants were reviewed and approved by the Ethics Committee of Fuzhou Children’s Hospital of Fujian Medical University. Written informed consent to participate in this study was provided by the participants’ legal guardian/next of kin.

## Author Contributions

XY drafted the initial manuscript. RC conceptualized and designed the study, and reviewed and revised the manuscript. YZ and XHY collected cases. XL did the laboratory testing. KM revised the manuscript and assisted with data management. All authors contributed to the article and approved the submitted version.

## Funding

This study was supported by Technology Innovation Team Train Project of Fuzhou Health Committee in China (2016-S-wp1), and sponsored by key Clinical Specialty Discipline Construction Program of Fuzhou, Fujian, P.R.C (201610191) and Fuzhou Children’s Medical Center (2018080310).

## Conflict of Interest

The authors declare that the research was conducted in the absence of any commercial or financial relationships that could be construed as a potential conflict of interest.
