# Supplementary material for: Gut Microbiota of Chinese Obese Children and Adolescents With and Without Insulin Resistance
Source: Front Endocrinol (Lausanne). 2021 Mar 19;12:636272. doi: 10.3389/fendo.2021.636272 (PMC8018175; doi:10.3389/fendo.2021.636272)
Supplement: Supplementary file 1 [file Table_1.docx]

**Table S1** Comparison of alpha-diversity between IR and IS subjects

| alpha-diversity index | H | P value |
| --- | --- | --- |
| Shannon | 0.012 | 0.914 |
| Observed OTUs | 1.179 | 0.278 |
| Faith’s phylogenetic diversity | 1.539 | 0.215 |
| Pielou’s evenness | 0.198 | 0.656 |

**Table S2.** Discriminant analysis table based on statistically different OTUs at phyla level in IR and IS subjects

| **predicted group membership** | | | | | |
| --- | --- | --- | --- | --- | --- |
|  |  | group | IR | IS | total |
| Original | count | IR | 34 | 6 | 40 |
|  |  | IS | 11 | 14 | 25 |
|  | % | IR | 85.0 | 15.0 | 100.0 |
|  |  | IS | 44.0 | 56.0 | 100.0 |

**Table S3.** Discriminant analysis table based on statistically different OTUs at phyla level in IR and IS subjects

| **predicted group membership** | | | | | |
| --- | --- | --- | --- | --- | --- |
|  |  | group | IR | IS | total |
| Original | count | IR | 37 | 3 | 40 |
|  |  | IS | 6 | 19 | 25 |
|  | % | IR | 92.5 | 7.5 | 100.0 |
|  |  | IS | 24.0 | 76.0 | 100.0 |
| Cross-validated | count | IR | 35 | 5 | 40 |
|  |  | IS | 7 | 18 | 25 |
|  | % | IR | 87.5 | 12.5 | 100.0 |
|  |  | IS | 28.0 | 72.0 | 100.0 |

**Table S4.** KEGGs biomarkers in IR and IS subjects.

| pathway | IR: mean rel. freq. (%) | IR: std. dev. (%) | IS: mean rel. freq. (%) | IS: std. dev. (%) | p-values |
| --- | --- | --- | --- | --- | --- |
| adenosylcobalamin biosynthesis from cobyrinate a,c-diamide I | 0.57 | 0.11 | 0.67 | 0.09 | 0.00 |
| 5-aminoimidazole ribonucleotide biosynthesis I | 0.75 | 0.11 | 0.81 | 0.06 | 0.03 |
| 5-aminoimidazole ribonucleotide biosynthesis II | 0.74 | 0.12 | 0.80 | 0.07 | 0.03 |
| 8-amino-7-oxononanoate biosynthesis I | 0.34 | 0.15 | 0.25 | 0.19 | 0.04 |
| adenosine ribonucleotides de novo biosynthesis | 0.80 | 0.13 | 0.89 | 0.08 | 0.01 |
| adenosylcobalamin salvage from cobinamide I | 0.60 | 0.10 | 0.69 | 0.09 | 0.00 |
| adenosylcobalamin salvage from cobinamide II | 0.58 | 0.11 | 0.67 | 0.09 | 0.00 |
| biotin biosynthesis I | 0.35 | 0.14 | 0.26 | 0.18 | 0.04 |
| Calvin-Benson-Bassham cycle | 0.84 | 0.11 | 0.90 | 0.08 | 0.04 |
| CDP-diacylglycerol biosynthesis I | 0.80 | 0.11 | 0.89 | 0.10 | 0.00 |
| CDP-diacylglycerol biosynthesis II | 0.80 | 0.11 | 0.89 | 0.10 | 0.00 |
| chorismate biosynthesis from 3-dehydroquinate | 0.75 | 0.10 | 0.82 | 0.07 | 0.01 |
| chorismate biosynthesis I | 0.75 | 0.10 | 0.83 | 0.08 | 0.00 |
| cis-vaccenate biosynthesis | 0.84 | 0.12 | 0.91 | 0.09 | 0.02 |
| coenzyme A biosynthesis I | 0.67 | 0.11 | 0.73 | 0.07 | 0.03 |
| colanic acid building blocks biosynthesis | 0.36 | 0.08 | 0.31 | 0.10 | 0.04 |
| D-galacturonate degradation I | 0.37 | 0.09 | 0.30 | 0.13 | 0.02 |
| flavin biosynthesis I (bacteria and plants) | 0.62 | 0.10 | 0.67 | 0.08 | 0.04 |
| guanosine ribonucleotides de novo biosynthesis | 0.71 | 0.11 | 0.78 | 0.07 | 0.01 |
| inosine-5'-phosphate biosynthesis I | 0.73 | 0.13 | 0.80 | 0.08 | 0.04 |
| L-arginine biosynthesis I (via L-ornithine) | 0.59 | 0.09 | 0.69 | 0.11 | 0.00 |
| L-arginine biosynthesis II (acetyl cycle) | 0.60 | 0.10 | 0.70 | 0.14 | 0.00 |
| L-arginine biosynthesis III (via N-acetyl-L-citrulline) | 0.38 | 0.11 | 0.29 | 0.14 | 0.01 |
| L-arginine biosynthesis IV (archaebacteria) | 0.59 | 0.09 | 0.69 | 0.11 | 0.00 |
| L-histidine biosynthesis | 0.66 | 0.13 | 0.73 | 0.09 | 0.03 |
| L-isoleucine biosynthesis I (from threonine) | 0.82 | 0.12 | 0.88 | 0.08 | 0.03 |
| L-isoleucine biosynthesis II | 0.88 | 0.12 | 0.94 | 0.09 | 0.04 |
| L-isoleucine biosynthesis III | 0.76 | 0.11 | 0.82 | 0.08 | 0.02 |
| L-isoleucine biosynthesis IV | 0.81 | 0.12 | 0.88 | 0.09 | 0.02 |
| L-lysine biosynthesis III | 0.79 | 0.11 | 0.85 | 0.07 | 0.02 |
| L-lysine biosynthesis VI | 0.79 | 0.11 | 0.84 | 0.07 | 0.03 |
| L-ornithine biosynthesis | 0.57 | 0.10 | 0.64 | 0.12 | 0.02 |
| L-tryptophan biosynthesis | 0.63 | 0.12 | 0.70 | 0.12 | 0.02 |
| L-valine biosynthesis | 0.82 | 0.12 | 0.88 | 0.08 | 0.03 |
| lipid IVA biosynthesis | 0.33 | 0.12 | 0.26 | 0.12 | 0.03 |
| methylerythritol phosphate pathway I | 0.73 | 0.13 | 0.80 | 0.08 | 0.03 |
| methylerythritol phosphate pathway II | 0.73 | 0.13 | 0.80 | 0.08 | 0.03 |
| O-antigen building blocks biosynthesis (E. coli) | 0.49 | 0.09 | 0.56 | 0.08 | 0.00 |
| palmitate biosynthesis II (bacteria and plants) | 0.30 | 0.22 | 0.19 | 0.13 | 0.04 |
| peptidoglycan biosynthesis I (meso-diaminopimelate containing) | 0.71 | 0.11 | 0.77 | 0.08 | 0.02 |
| peptidoglycan biosynthesis III (mycobacteria) | 0.71 | 0.11 | 0.77 | 0.08 | 0.01 |
| peptidoglycan biosynthesis IV (Enterococcus faecium) | 0.13 | 0.11 | 0.19 | 0.14 | 0.04 |
| phosphopantothenate biosynthesis I | 0.56 | 0.09 | 0.61 | 0.10 | 0.03 |
| pyridoxal 5'-phosphate biosynthesis I | 0.13 | 0.09 | 0.09 | 0.07 | 0.03 |
| pyruvate fermentation to isobutanol (engineered) | 0.90 | 0.11 | 0.97 | 0.11 | 0.02 |
| sulfate reduction I (assimilatory) | 0.13 | 0.09 | 0.09 | 0.06 | 0.03 |
| superpathway of 5-aminoimidazole ribonucleotide biosynthesis | 0.74 | 0.12 | 0.80 | 0.07 | 0.03 |
| superpathway of adenosine nucleotides de novo biosynthesis I | 0.77 | 0.10 | 0.83 | 0.06 | 0.01 |
| superpathway of aromatic amino acid biosynthesis | 0.79 | 0.10 | 0.87 | 0.09 | 0.00 |
| superpathway of branched amino acid biosynthesis | 0.77 | 0.11 | 0.83 | 0.08 | 0.02 |
| superpathway of fucose and rhamnose degradation | 0.20 | 0.10 | 0.14 | 0.06 | 0.01 |
| superpathway of geranylgeranyl diphosphate biosynthesis II (via MEP) | 0.70 | 0.13 | 0.77 | 0.08 | 0.03 |
| superpathway of heme biosynthesis from glycine | 0.01 | 0.01 | 0.02 | 0.02 | 0.04 |
| superpathway of hexitol degradation (bacteria) | 0.22 | 0.13 | 0.13 | 0.09 | 0.01 |
| superpathway of hexuronide and hexuronate degradation | 0.25 | 0.09 | 0.21 | 0.07 | 0.05 |
| superpathway of L-isoleucine biosynthesis I | 0.78 | 0.10 | 0.83 | 0.07 | 0.04 |
| superpathway of menaquinol-8 biosynthesis II | 0.10 | 0.11 | 0.05 | 0.05 | 0.04 |
| superpathway of phospholipid biosynthesis I (bacteria) | 0.74 | 0.08 | 0.80 | 0.09 | 0.01 |
| superpathway of pyridoxal 5'-phosphate biosynthesis and salvage | 0.17 | 0.11 | 0.12 | 0.08 | 0.03 |
| superpathway of pyrimidine deoxyribonucleosides degradation | 0.46 | 0.08 | 0.52 | 0.14 | 0.04 |
| superpathway of pyrimidine nucleobases salvage | 0.83 | 0.13 | 0.92 | 0.10 | 0.01 |
| superpathway of sulfate assimilation and cysteine biosynthesis | 0.22 | 0.10 | 0.16 | 0.09 | 0.02 |
| superpathway of tetrahydrofolate biosynthesis | 0.53 | 0.07 | 0.48 | 0.12 | 0.04 |
| superpathway of UDP-glucose-derived O-antigen building blocks biosynthesis | 0.13 | 0.08 | 0.09 | 0.06 | 0.03 |
| thiamin salvage II | 0.58 | 0.11 | 0.66 | 0.11 | 0.01 |
| tRNA charging | 0.70 | 0.10 | 0.75 | 0.07 | 0.02 |
| UDP-N-acetyl-D-glucosamine biosynthesis I | 0.44 | 0.09 | 0.55 | 0.13 | 0.00 |
| UDP-N-acetylmuramoyl-pentapeptide biosynthesis I (meso-diaminopimelate containing) | 0.72 | 0.11 | 0.78 | 0.08 | 0.01 |
| UDP-N-acetylmuramoyl-pentapeptide biosynthesis II (lysine-containing) | 0.72 | 0.11 | 0.79 | 0.08 | 0.01 |
| UMP biosynthesis | 0.79 | 0.12 | 0.85 | 0.09 | 0.03 |
